# Metabolic syndrome is associated with gastroesophageal reflux disease in a large Taiwanese population study

**DOI:** 10.7150/ijms.109616

**Published:** 2025-02-28

**Authors:** Wen-Hung Hsu, Jiun-Hung Geng, Pei-Yu Wu, Jiun-Chi Huang, Chao-Hung Kuo, Szu-Chia Chen

**Affiliations:** 1 Department of Internal Medicine, Kaohsiung Municipal Siaogang Hospital, Kaohsiung Medical University Hospital, Kaohsiung Medical University, Kaohsiung 812, Taiwan.; 2 Division of Gastroenterology, Department of Internal Medicine, Kaohsiung Medical University Hospital, Kaohsiung Medical University, Kaohsiung 807, Taiwan.; 3 Department of Urology, Kaohsiung Municipal Siaogang Hospital, Kaohsiung Medical University, Kaohsiung, Taiwan.; 4 Department of Urology, Kaohsiung Medical University Hospital, Kaohsiung Medical University, Kaohsiung, Taiwan.; 5 Division of Nephrology, Department of Internal Medicine, Kaohsiung Medical University Hospital, Kaohsiung Medical University, Kaohsiung 807, Taiwan.; 6 Faculty of Medicine, College of Medicine, Kaohsiung Medical University, Kaohsiung 807, Taiwan.

**Keywords:** metabolic syndrome, gastroesophageal reflux disease, Taiwan Biobank

## Abstract

Gastroesophageal reflux disease (GERD) and metabolic syndrome (MetS) have emerged as prominent health issues in past decades. This study aimed to evaluate whether the presence of MetS and its constituent factors was associated with a higher likelihood of GERD in a large cohort of Taiwanese adults. MetS and its components were defined according to the modified National Cholesterol Education Program Expert Panel and Adult Treatment Panel III for Asians, and the presence of GERD was assessed using standardized interviews and questionnaires. Of 121,583 participants from the Taiwan biobank, 16,664 (13.7%) were diagnosed with GERD and 27,441 (22.6%) were diagnosed with MetS. After multivariable analysis, the participants with MetS (odds ratio [OR] = 1.079), abdominal obesity (OR = 1.094), hypertriglyceridemia (OR = 1.085), and low high-density lipoprotein cholesterol (OR = 1.073) (all *p* < 0.001) were significantly associated with GERD, but high blood pressure and hyperglycemia were not. Furthermore, there was a trend of a stepwise increase in the rate of GERD in accordance with the number of MetS components. Participants with 3 components (*vs.* 0; OR = 1.093; *p* = 0.002), 4 components (*vs.* 0; OR = 1.108; *p* = 0.005), and 5 components (*vs.* 0; OR = 1.137; *p* = 0.029) were significantly associated with GERD. Our results suggest that MetS may be associated with the development of GERD in the Taiwanese population.

## Introduction

Gastroesophageal reflux disease (GERD) is an increasingly significant health issue in Asian countries, resulting in significant morbidity and substantial burden on medical expenditure 1. The adoption of western lifestyle habits in Asian countries has led to an increasing prevalence of both GERD and metabolic syndrome (MetS) 2, 3. Reports indicate that in Taiwan, the prevalence of reflux esophagitis has increased from 9% to 25% in the past decades 4-6. Risk factors for reflux esophagitis include older age, sex, obesity, smoking, and the consumption of alcohol and fatty foods 7-12, of which obesity is the most significant 13, 14. While obesity is a major risk factor for reflux esophagitis, it is noteworthy that GERD may have multiple pathogenic mechanisms, and not all of them may be linked to or influenced by obesity 15. The precise pathophysiological mechanisms underpinning this association have yet to be completely elucidated. Further evidence suggests that abdominal obesity, a fundamental component of MetS, may also be an important risk factor for reflux esophagitis 16, 17. MetS is defined by the presence of metabolic abnormalities including high triglyceride level, abdominal obesity, hyperglycemia, hypertension, and low level of high-density lipoprotein cholesterol (HDL-C), and it has emerged as a prominent health issue in recent decades 18, 19. MetS is closely tied to environmental conditions and diet behavior 20. The dramatic increase in both MetS and GERD has sparked research into the underlying pathophysiology. Given the increasing importance of these conditions and their effect on public health, we evaluated the associations between MetS and its five components with GERD in this study of enrollees in the Taiwan Biobank (TWB). Our aim was to evaluate whether MetS and its components were associated with an increased likelihood of GERD.

## Materials and methods

The TWB was established by the Ministry of Health and Welfare and approved by the Institutional Review Board on Biomedical Science Research, Academia Sinica, Taiwan, and the Ethics and Governance Council of the TWB. Established in 2008 as a large-scale community-based re-search program, the TWB database includes the data from Taiwanese adults aged 30 to 70 years and has been used in previous studies to explore the connections between clinical, personal health factors and various diseases 21-26. Eligible participants were required to be of Taiwanese ethnicity, provide written informed consent, and have no history of severe chronic diseases such as cancer at enrollment. Individuals with incomplete baseline data were excluded. Participants were selected based on their willingness to provide biospecimens and completed questionnaires regarding lifestyle, medical history, and other health-related factors.

### Study participants

The study participants between 2008 to 2016 were enrolled from the TWB. According to the inclusion criteria of the TWB, none of the participants had a history of cancer, and they were all aged from 30 to 70 years. Information included in the TWB covered lifestyle factors, genetic data and medical history 26, 27. All of the participants were interviewed and underwent physical examinations, during which fasting blood samples were obtained. The baseline study included 121,583 participants.

### Study variables

Blood pressure (BP: systolic and diastolic), weight, height, waist and hip circumference were recorded. The presence of hypertension and diabetes mellitus was also recorded, along with exercise habits, sex and age. In this study, we considered regular exercise as playing a sport or engaging in a physical activity other than work-related on at least 3 occasions in 1 week with each session lasting 30 minutes or more.

Laboratory tests were conducted using the fasting blood specimens to obtain data on uric acid, glucose, hemoglobin, triglycerides, total cholesterol, HDL-C, low-density lipoprotein cholesterol (LDL-C), and estimated glomerular filtration rate (eGFR: calculated according to the method proposed by Lewy et al 28).

### Definitions of GERD and MetS

The participants were asked, “Have you been diagnosed with GERD?”, and those who replied “Yes” were considered to have GERD. The participants were defined as having MetS if they had ≥ 3 of these criteria following the modified criteria for Asians 4 and National Cholesterol Education Program Expert Panel and Adult Treatment Panel III (NCEP-ATP III) guidelines 4: (1) hypertension, defined as systolic/diastolic BP ≥ 130/85 mmHg or antihypertensive agent prescriptions; (2) triglycerides ≥ 150 mg/dL; (3) HDL-C in women/men of < 50/40 mg/dL; (4) abdominal obesity, defined as a waist circumference in women/men of ≥ 80/90 cm, respectively; and (5) hyperglycemia, defined as a fasting glucose concentration ≥ 100 mg/dL or a diagnosis of diabetes mellitus.

### Statistical analysis

Continuous data are given as mean (SD) and compared with the independent t test. Categorical data are given as number (%) and compared with the chi-square test. Univariable analysis was performed, and significant variables were then entered into multivariable logistic regression analysis to evaluate associations among MetS and its components with GERD. SPSS version 19.0 (IBM Inc., Armonk, New York, USA) was used for the analysis, and two-tailed *p*-values < 0.05 were considered significant.

## Results

The 121,583 enrolled participants had a mean age of 49.9 ± 11.0 years, 43,698 were men and 77,885 were women. In addition, 16,664 (13.7%) had GERD and 104,919 (86.3%) did not.

### Clinical characteristics of the GERD groups

A comparison of the clinical characteristics between the participants without or with GERD is shown in Table 1. Compared to the participants without GERD (n = 104,919), those with GERD (n = 16,664) were older (49.6 *vs.* 51.9 years, *p* < 0.001), predominantly female (65.8 *vs.* 63.8%, *p* < 0.001), had higher prevalence rates of diabetes mellitus (6.3 *vs.* 5.0%, *p* < 0.001) and hypertension (15.3 *vs.* 11.7%, *p* < 0.001), higher prevalence rates of smoking (29.3 *vs.* 26.9%, *p* < 0.001) and alcohol history (9.8 *vs.* 8.3%, *p* < 0.001), higher prevalence of regular exercise habit (43.0 *vs.* 40.2%, *p* < 0.001), lower height (161.5 *vs.* 162.0 cms, *p* < 0.001), lower weight (63.5 *vs.* 63.8 kg, *p* = 0.010), higher waist circumference (83.7 *vs.* 83.2 cm, *p* < 0.001), lower hip circumference (95.9 *vs.* 96.0 cm, *p* = 0.011), higher fasting glucose (96.4 *vs.* 95.9 mg/dL, *p* = 0.002), higher triglyceride (119.0 *vs.* 115.1 mg/dL, *p* < 0.001), higher total cholesterol (197.4 *vs.* 195.4 mg/dL, *p* < 0.001), higher LDL-C (121.8 *vs.* 120.8 mg/dL, *p* < 0.001), lower eGFR (102.1 *vs.* 103.5 mL/min/1.73m^2^, *p* < 0.001) and lower uric acid (5.39 *vs.* 5.43 mg/dL, *p* = 0.002). Regarding MetS, the participants with GERD had higher prevalence of MetS (24.9 *vs.* 22.2%, *p* < 0.001), higher numbers of MetS components (1.6 *vs.* 1.5, *p* < 0.001), higher prevalence of five components, including abdominal obesity (49.4 *vs.* 46.0%, *p* < 0.001), hypertriglyceridemia (22.4 *vs.* 20.7%, *p* < 0.001), low HDL-cholesterol (26.6 *vs.* 25.4%, *p* = 0.001), hyperglycemia (22.7 *vs.* 20.5%, *p* < 0.001) and high BP (37.1 *vs.* 34.9%, *p* < 0.001).

### Association of MetS and GERD

The factors associated with GERD in the study participants in multivariable logistic regression analysis are shown in Table 2. The correlation matrix in the logistic regression model reveals a strong correlation (|r| = 0.87) between LDL-cholesterol and total cholesterol (supplementary Table 1), indicating potential multicollinearity. In the linear regression model, the Variance Inflation Factor (VIF) analysis showed that total cholesterol and LDL-cholesterol had a relatively high VIF of approximately 4.39 (supplementary Table 2). Therefore, we do not put LDL-cholesterol in the further multivariable analysis.

After adjusting for age, sex, smoking and alcohol history, regular exercise habit, total cholesterol, eGFR, uric acid and MetS (significant variables in univariable analysis of Table 1), MetS (odds ratio [OR] = 1.079; 95% confidence interval [CI] = 1.037-1.124; *p* < 0.001), old age (*p* < 0.001), female (*p* < 0.001), smoking history (*p* < 0.001), alcohol history (*p* < 0.001), high total cholesterol (*p* = 0.021), low eGFR (*p* = 0.025), and low uric acid (*p* < 0.001) remained significant factors for GERD in the multivariable logistic regression.

### Association of number of MetS components and GERD

The associations among the number of MetS components and GERD in multivariable logistic regression analysis are shown in Table 3. After multivariable analysis adjusting for age, sex, smoking and alcohol history, regular exercise habit, total cholesterol, LDL-cholesterol, eGFR and uric acid, participants with 3 MetS components (*vs.* 0; OR = 1.093; 95% CI = 1.033-1.157; *p* = 0.002), 4 MetS components (*vs.* 0; OR = 1.108; 95% CI = 1.031-1.190; *p* = 0.005), and 5 MetS components (*vs.* 0; OR = 1.137; 95% CI = 1.073-1.275; *p* = 0.029) were significantly associated with GERD.

### Association of MetS components and GERD

The associations among the MetS components and GERD in multivariable logistic regression analysis are shown in Table 4. After multivariable analysis adjusting for age, sex, smoking and alcohol history, regular exercise habit, total cholesterol, LDL-cholesterol, eGFR and uric acid, participants with abdominal obesity (OR = 1.094; 95% CI = 1.057-1.132; *p* < 0.001), hypertriglyceridemia (OR = 1.085; 95% CI = 1.040-1.131; *p* < 0.001), and low HDL-cholesterol (OR = 1.073; 95% CI = 1.033-1.116; *p* < 0.001), were significantly associated with GERD. However, hyperglycemia (*p* = 0.532), and high BP (*p* = 0.062) were not associated with GERD.

## Discussion

In this study of associations between MetS and its components with GERD in 121,583 participants from the TWB, we found the MetS group was associated with a high prevalence of GERD. Furthermore, of the MetS components, associations were found between hypertriglyceridemia, abdominal obesity, and low HDL-C with a high risk of GERD, but hyperglycemia and hypertension were not.

There are three key findings. First, the association between MetS and GERD, as well as the trend of a stepwise increase in the prevalence of GERD in accordance with the number of MetS components. MetS and its components abdominal obesity, hyperglycemia, hypertension and dyslipidemia predispose individuals to diabetes and cardiovascular disease 29, 30. Its fundamental pathological mechanisms involve obesity and insulin resistance. The NCEP-ATP III guidelines reported that the overall prevalence of MetS is approximately 15% to 23%, escalating significantly with age 19, 31. Previous studies based on endoscopic examination findings and classification of reflux esophagitis have delineated a close relationship between reflux esophagitis and MetS 32-34. A cross-sectional study conducted in South Korean in 2008 included 1679 patients with erosive esophagitis and found that 21% of the patients had MetS, which was much higher than the control group (12%) 32. In Taiwan, a retrospective study conducted in 2016 showed that 20% (19/95) of patients with Barrett's esophagus and 9.6% (214/1118) of those with erosive gastritis had MetS 33. Similar findings were also reported in cross-sectional study of 4859 patients in 2019. Among 2916 patients with erosive esophagitis, 949 (32.5%) had MetS, which was much higher than the control group (22.5%; 446/1079) 34. Most of these studies evaluated endoscopic erosive esophagitis, but excluded non-erosive esophagitis. Although the mechanism of the association between erosive esophagitis and MetS has yet to be elucidated, several possible factors have been proposed. An independent association between increased waist circumference, one of the most important components of MetS, and an elevated risk of erosive esophagitis has been reported. In addition, visceral fat accumulation can increase intra-abdominal pressure and promote gastroesophageal reflux 35. Moreover, hypertriglyceridemia may be associated with a high-fat diet, which can result in delayed gastric emptying and further promote esophagogastric reflux 36. Hypertension has also been considered to be a significant risk factor for erosive esophagitis due to the esophageal sphincter pressure-lowering effect of antihypertensive medications such as calcium channel blockers, and environmental factors such as life stress and high-salt diet have been shown to promote esophagogastric reflux 37, 38. Hyperglycemia has also been associated with erosive esophagitis, possibly due to diabetic autonomic neuropathy and insulin resistance. The association between HDL-C and erosive esophagitis is controversial in previous studies. The present study of 121,583 participants is the largest population-based investigation to date. Of these participants, 16,664 (13.7%) were diagnosed with GERD and 27,441 (22.6%) were diagnosed with MetS. Notably, we found a correlation between the number of MetS components and the presence of reflux esophagitis, including erosive esophagitis and non-erosive esophagitis. A higher number of MetS components signifies not only a relationship between the two conditions, but also suggests a certain level of interaction.

The association between abdominal obesity and GERD is the second important finding of this study. Abdominal obesity has been hypothesized to promote gastroesophageal reflux through an intra-abdominal pressure-increasing effect and transient relaxation of the lower esophageal sphincter 19, 39. Beyond mechanical causes, adipose tissue has been demonstrated to stimulate the overproduction of pro-inflammatory cytokines, leading to low esophageal sphincter relaxation and insulin resistance 40, 41.

The third key finding is the relationship between hypertriglyceridemia and low HDL-C with GERD. Hypertriglyceridemia has been associated with high dietary fat intake, and a diet overly high in fat diet can lead to delayed gastric emptying and further increase the risk of GERD 36, 41. In this study, we found a weak association between low HDL-C and GERD. Associations between many diseases and a change in HDL-C level and composition have been reported. In addition, a decreased HDL-C level has been observed in patients with acute and chronic inflammatory diseases such as ankylosing spondylitis 42. These findings suggest that a change in lipid metabolism may be the result of both MetS and GERD.

We also found that hyperglycemia and high BP were not associated with GERD. Hyperglycemia is caused by changes in obesity-related hormones and insulin resistance, which have been associated with an increased prevalence of erosive esophagitis and GERD symptoms 43. The association between hyperglycemia with reflux esophagitis remains controversial 32, 36. While some studies have hypothesized that hyperglycemia may contribute to reflux esophagitis through diabetic autonomic neuropathy and insulin resistance 40, 41, this phenomenon was not found in our study. Delayed gastric emptying associated with diabetic autonomic neuropathy may represent a late stage of hyperglycemia, which was not evident in this study population. Hypertension has been associated with erosive esophagitis, but again we did not observe this in the present study. Since this is a cross-sectional study and due to the nature of the data stored in the TWB, we could not analyze environmental factors and drug history.

In our study, the results revealed that female sex is an independent risk factor for total GERD. TWB conducted wider spectrum epidemiologic study for reflux esophagitis including erosive esophagitis and non-erosive esophagitis. Although many studies showed male sex is a risk factor for erosive esophagitis 9, 10, several studies demonstrated female sex is associated with non-erosive esophagitis 11, 12. TWB collects health-related data on healthy volunteers around Taiwan, women may be more willing or able to participate in research studies compared to men due to higher health awareness. As the presence of GERD was checked through the use of questionnaires without endoscopic verification, the severity and type of GERD could not be verified. Above explanations may partially explain the difference.

The key advantages of our research include the large Taiwanese adult cohort drawn from the community, and that we controlled for a wide range of confounding variables spanning lifestyle and health-related factors. Nevertheless, several limitations should also be mentioned. First, we lacked information about medications used to treat the various components of MetS. As these agents may have had an effect on the occurrence and prevention of MetS and its components, the associations between MetS and its components with GERD may be underestimated. Second, the cross-sectional design of the study meant that were unable to evaluate the duration of GERD, and hence we could not analyze causal relationships between MetS, its components and GERD was not possible. Longitudinal studies may be able to overcome this limitation. Third, as the presence of GERD was checked through the use of questionnaires without endoscopic verification, the severity and type of GERD could not be verified. However, a previous study in Taiwan showed moderate agreement between claims data and self-reported diseases 44. The final limitation is that the study participants were of Chinese ethnicity, thus potentially limiting the conclusions to other ethnicities.

In conclusion, the results of this large cohort study of Taiwanese adults showed associations between MetS and its components abdominal obesity, hypertriglyceridemia, and low HDL-C with a high prevalence of GERD. Further, there was a trend of a stepwise increase in the prevalence of GERD in accordance with the number of MetS components. These results may indicate that MetS plays a role in the risk of GERD in the Taiwanese population. Further longitudinal studies are warranted to investigate the risk of incident GERD, and whether improving MetS can reduce the incidence of GERD.

## Supplementary Material

Supplementary tables.

## Figures and Tables

**Table 1:** Comparison of clinical characteristics among participants according to GERD in study participants

Characteristics	GERD (-) (*n* = 104,919)	GERD (+) (*n* = 16,664)	*p*
Age (year)	49.6 ± 11.0	51.9 ± 10.4	< 0.001
Male sex (%)	36.2	34.2	< 0.001
DM (%)	5.0	6.3	< 0.001
Hypertension (%)	11.7	15.3	< 0.001
Smoking history (%)	26.9	29.3	< 0.001
Alcohol history (%)	8.3	9.8	< 0.001
Regular exercise habits (%)	40.2	43.0	< 0.001
Systolic BP (mmHg)	120.4 ± 18.8	120.7 ± 17.8	0.102
Diastolic BP (mmHg)	73.8 ± 11.5	73.8 ± 10.9	0.562
Body height (cm)	162.0 ± 8.3	161.5 ± 8.2	< 0.001
Body weight (kg)	63.8 ± 12.7	63.5 ± 12.7	0.010
Waist circumference (cm)	83.2 ± 10.2	83.7 ± 10.3	< 0.001
Hip circumference (cm)	96.0 ± 7.1	95.9 ± 7.2	0.011
Laboratory parameters			
Fasting glucose (mg/dL)	95.9 ± 20.9	96.4 ± 19.7	0.002
Hemoglobin (g/dL)	13.8 ± 1.6	13.8 ± 1.5	0.083
Triglyceride (mg/dL)	115.1 ± 94.3	119.0 ± 92.3	< 0.001
Total cholesterol (mg/dL)	195.4 ± 35.9	197.4 ± 35.2	< 0.001
HDL-C (mg/dL)	54.6 ± 13.4	54.6 ± 13.6	0.668
LDL-C (mg/dL)	120.8 ± 31.8	121.8 ± 31.3	< 0.001
eGFR (mL/min/1.73 m^2^)	103.5 ± 23.9	102.1 ± 23.7	< 0.001
Uric acid (mg/dL)	5.43 ± 1.43	5.39 ± 1.39	0.002
MetS (%)	22.2	24.9	< 0.001
Number of MetS components	1.5 ± 1.3	1.6 ± 1.3	< 0.001
MetS component			
Abdominal obesity (%)	46.0	49.4	< 0.001
Hypertriglyceridemia (%)	20.7	22.4	< 0.001
Low HDL-cholesterol (%)	25.4	26.6	0.001
Hyperglycemia (%)	20.5	22.7	< 0.001
High blood pressure (%)	34.9	37.1	< 0.001

Abbreviations. GERD, gastroesophageal reflux disease; DM, diabetes mellitus; BP, blood pressure; HDL-C, high-density lipoprotein cholesterol; LDL-C, low-density lipoprotein cholesterol; eGFR, estimated glomerular filtration rate; MetS, metabolic syndrome.

**Table 2:** Risks of GERD using multivariable logistic regression analysis

Variables	Multivariable (GERD)	
	Odds ratio (95% CI)	*p*
Age (per 1 year)	1.019 (1.018-1.021)	< 0.001
Male *vs.* female	0.819 (0.782-0.859)	< 0.001
Smoking history	1.253 (1.199-1.310)	< 0.001
Alcohol history	1.176 (1.108-1.249)	< 0.001
Regular exercise habits	0.982 (0.948-1.018)	0.323
Total cholesterol (per 10 mg/dL)	1.005 (1.001-1.010)	0.021
eGFR (per 1 mL/min/1.73 m^2^)	0.999 (0.998-1.000)	0.025
Uric acid (per 1 mg/dL)	0.968 (0.954-0.982)	< 0.001
MetS	1.079 (1.037-1.124)	< 0.001

Values expressed as odds ratio and 95% confidence interval (CI). Abbreviations are the same as in Table 1. Adjusted for age, sex, smoking and alcohol history, regular exercise habit, total cholesterol, eGFR, uric acid and MetS.

**Table 3:** Association of the number of MetS components and GERD using multivariable logistic regression analysis

Number of MetS components	Multivariable (GERD)	
	Odds ratio (95% CI)	*p*
0 MetS component	Reference	
1 MetS component	1.014 (0.968-1.061)	0.558
2 MetS components	1.042 (0.992-1.096)	0.100
3 MetS components	1.093 (1.033-1.157)	0.002
4 MetS components	1.108 (1.031-1.190)	0.005
5 MetS components	1.137 (1.073-1.275)	0.029

Values expressed as odds ratio and 95% confidence interval (CI). Abbreviations are the same as in Table 1. Adjusted for age, sex, smoking and alcohol history, regular exercise habit, total cholesterol, eGFR, uric acid and the number of MetS components.

**Table 4:** Association of MetS components and GERD using multivariable logistic regression analysis

MetS components	Multivariable (GERD)	
	Odds ratio (95% CI)	*p*
Abdominal obesity	1.094 (1.057-1.132)	< 0.001
Hypertriglyceridemia	1.085 (1.040-1.131)	< 0.001
Low HDL-cholesterol	1.073 (1.033-1.116)	< 0.001
Hyperglycemia	1.013 (0.972-1.056)	0.532
High blood pressure	0.965 (0.930-1.002)	0.062

Values expressed as odds ratio and 95% confidence interval (CI). Abbreviations are the same as in Table 1. Adjusted for age, sex, smoking and alcohol history, regular exercise habit, total cholesterol, eGFR, uric acid and MetS component.
