# Supplementary material for: Metabolic syndrome is associated with gastroesophageal reflux disease in a large Taiwanese population study
Source: Int J Med Sci. 2025 Feb 28;22(7):1555–61. doi: 10.7150/ijms.109616 (PMC11905277; doi:10.7150/ijms.109616)
Supplement: Supplementary file 1 — Supplementary tables. [file ijmsv22p1555s1.pdf]

Supplementary Table 1. The Correlation Matrix

Parameter	Intercept	Age (per 1 year)	Male *vs.* female	Smoking history	Alcohol history	Regular exercise habits	Total cholesterol (per 10 mg/dL)	LDL-cholesterol (per 1 mg/dL)	eGFR (per 1 mL/min/1.73 m^2)	Uric acid (per 1 mg/dL)	MetS
Intercept	1.00	-0.44	0.10	-0.21	-0.20	-0.13	-0.33	0.15	-0.45	-0.67	0.14
Age (per 1 year)	-0.44	1.00	0.00	0.05	-0.02	-0.31	-0.09	0.03	0.07	0.26	-0.18
Male *vs.* female	0.10	0.00	1.00	-0.43	-0.12	-0.03	0.17	-0.12	-0.37	0.12	0.06
Smoking history	-0.21	0.05	-0.43	1.00	-0.22	0.04	0.00	-0.01	0.00	0.00	-0.03
Alcohol history	-0.20	-0.02	-0.12	-0.22	1.00	-0.01	-0.08	0.09	-0.05	-0.03	-0.02
Regular exercise habits	-0.13	-0.31	-0.03	0.04	-0.01	1.00	-0.03	0.04	-0.01	0.03	0.07
Total cholesterol (per 10 mg/dL)	-0.33	-0.09	0.17	0.00	-0.08	-0.03	1.00	-0.87	0.01	0.05	-0.01
LDL-cholesterol (per 1 mg/dL)	0.15	0.03	-0.12	-0.01	0.09	0.04	-0.87	1.00	-0.07	-0.03	0.02
eGFR (per 1 mL/min/1.73 m^2)	-0.45	0.07	-0.37	0.00	-0.05	-0.01	0.01	-0.07	1.00	0.29	-0.25
Uric acid (per 1 mg/dL)	-0.67	0.26	0.12	0.00	-0.03	0.03	0.05	-0.03	0.29	1.00	-0.04
MetS	0.14	-0.18	0.06	-0.03	-0.02	0.07	-0.01	0.02	-0.25	-0.04	1.00

Values expressed as *r*.

Abbreviations. LDL-C, low-density lipoprotein cholesterol; eGFR, estimated glomerular filtration rate; MetS, metabolic syndrome.

Supplementary Table 2. The Variance Inflation Factor (VIF)

Variable	Odds ratio (95% CI)	*p*	VIF
Age (per 1 year)	1.019 (1.018-1.021)	< 0.001	1.30
Male *vs.* female	0.823 (0.785-0.863)	< 0.001	1.82
Smoking history	1.253 (1.199-1.310)	< 0.001	1.42
Alcohol history	1.171 (1.103-1.244)	< 0.001	1.15
Regular exercise habits	0.981 (0.947-1.017)	0.294	1.14
Total cholesterol (per 10 mg/dL)	1.012 (1.002-1.021)	0.015	4.39
LDL-cholesterol (per 1 mg/dL)	0.999 (0.998-1.000)	0.137	4.32
eGFR (per 1 mL/min/1.73 m^2)	0.999 (0.998-1.000)	0.028	1.34
Uric acid (per 1 mg/dL)	0.969 (0.955-0.983)	< 0.001	1.65
MetS	1.079 (1.036-1.123)	< 0.001	1.12

Abbreviations. LDL-C, low-density lipoprotein cholesterol; eGFR, estimated glomerular filtration rate; MetS, metabolic syndrome.
